# The *Shigella* Type Three Secretion System Effector OspG Directly and Specifically Binds to Host Ubiquitin for Activation

**DOI:** 10.1371/journal.pone.0057558

**Published:** 2013-02-28

**Authors:** Yan Zhou, Na Dong, Liyan Hu, Feng Shao

**Affiliations:** 1 College of Life Sciences, Beijing Normal University, Beijing, China; 2 National Institute of Biological Sciences, Beijing, China; University of Louisville, United States of America

## Abstract

The genus *Shigella* infects human gut epithelial cells to cause diarrhea and gastrointestinal disorders. Like many other Gram-negative bacterial pathogens, the virulence of *Shigella spp.* relies on a conserved type three secretion system that delivers a handful of effector proteins into host cells to manipulate various host cell physiology. However, many of the *Shigella* type III effectors remain functionally uncharacterized. Here we observe that OspG, one of the *Shigella* effectors, interacted with ubiquitin conjugates and poly-ubiquitin chains of either K48 or K63 linkage in eukaryotic host cells. Purified OspG protein formed a stable complex with ubiquitin but showed no interactions with other ubiquitin-like proteins. OspG binding to ubiquitin required the carboxyl terminal helical region in OspG and the canonical I44-centered hydrophobic surface in ubiquitin. OspG and OspG-homologous effectors, NleH1/2 from *enteropathogenic E coli* (EPEC), contain sub-domains I-VII of eukaryotic serine/threonine kinase. GST-tagged OspG and NleH1/2 could undergo autophosphorylation, the former of which was significantly stimulated by ubiquitin binding. Ubiquitin binding was also required for OspG functioning in attenuating host NF-κB signaling. Our data illustrate a new mechanism that bacterial pathogen like *Shigella* exploits ubiquitin binding to activate its secreted virulence effector for its functioning in host eukaryotic cells.

## Introduction

*Shigella spp.* are Gram-negative bacterial pathogens whose infection causes a spectrum of symptoms ranging from watery diarrhea to severe dysentery [1]. *Shigella* harbors a 220-kb virulence plasmid that is essential for successful infection [2]. This plasmid encodes a specialized protein secretion apparatus called the type three secretion system as well as more than a dozen of type III-secreted effector proteins. Like in many other Gram-negative bacterial pathogens, effector secreted by *Shigella* type III secretion system function to facilitate infection, bacterial survival and replication [3]. Studies in the past 20 years have shown that type III secreted effectors can target multiple host cellular processes such as innate immunity, actin cytoskeleton dynamics and membrane trafficking [3], [4]. In *Shigella* infection, the OspF effector functions as a novel MAPK phosphothreonine lyase to irreversibly inactivate mitogen-activated protein kinase (MAPK) [5] and downregulate host inflammatory responses [6], [7]. IpgB1/2 are guanine-nucleotide exchange factors (GEFs) and preferentially activate small GTPase Rac1 and RhoA, respectively, to promote *Shigella* invasion of host epithelial cells [8], [9], [10]. The VirA effector is a novel TBC-like GTPase-activating protein (GAP), which inactivates host Rab1 and contributes to *Shigella* escape from host autophagy [11]. Expression of these *Shigella* effectors is under sophisticated genetic regulation by temperature and contact host cell contact. However, little is known about the regulation of effector biochemical activities.

Protein ubiquitination critically controls nearly all aspects of eukaryotic cellular processes including cell cycle progression, gene transcription, various signal transduction pathways [12]. Ubiquitination involves a three-enzyme cascade composed of ubiquitin-activating enzyme, ubiquitin-conjugating enzyme and ubiquitin ligase. In the third step, ubiquitin ligase catalyzes ubiquitin transfer from the ubiquitin-conjugating enzyme onto a lysine side chain in the substrate or another ubiquitin linked through an isopeptide bond linkage. The latter generates either free ubiquitin chains or ubiquitin chains attached to a substrate protein. Ubiquitin conjugation onto one of the seven lysines in another ubiquitin results in formation of ubiquitin chains with different linkages that often confer different fates on the substrate protein [13]. The ubiquitin system is frequently hijacked by bacterial pathogens [14], [15], and bacterial type III effectors can even directly deamidate ubiquitin to paralyze its chain formation activity [16]. In the case of *Shigella* infection, the OspI effector deamidates an ubiquitin-conjugating enzyme Ubc13 that is dedicated to form K63-linked ubiquitin chains to dampen host NF-κB-mediated inflammatory response [17]. *Shigella* also harbors a group of effectors called IpaHs that define a third class of ubiquitin ligases structurally distinct from eukaryotic HECT- and RING-domain ubiquitin ligases [18], [19], [20]. IpaHs have also been implicated in tuning the host inflammatory responses [21].

OspG is another *Shigella* type III effector. OspG was firstly identified from the supernatant of *Shigella flexneri* Δ*ipaBCDA* strain that secretes effectors constitutively [22]. Expression of OspG is induced only after activation of the type three secretion apparatus, suggesting its function at a later stage during *Shigella* infection [23]. OspG shares sequence similarity to mammalian serine/threonine protein kinase and may interfere with innate immune responses by targeting host ubiquitin-conjugating enzyme [24]. However, the biochemical mechanism and regulation of OspG function remains largely unknown.

In this work, we discover that OspG binds to free ubiquitin, poly-ubiquitin chains as well as ubiquitin-conjugated proteins with high affinity and specificity. We further demonstrate that OspG binding to ubiquitin is mediated by the canonical I44-centered hydrophobic surface in ubiquitin and requires a unique carboxyl terminal helix in OspG. Ubiquitin binding significantly stimulates the kinase activity of OspG, shown by evidently increased autophosphorylation and intrinsic ATP hydrolysis activity. Ubiquitin binding is also required for OspG functioning in attenuating host innate immune NF-κB signaling. Our data provide the first example that bacterial effectors can make use of interaction with host ubiquitin to activate itself.

## Results

### OspG Directly Binds to Polyubiquitin Conjugates

**Figure 1:**
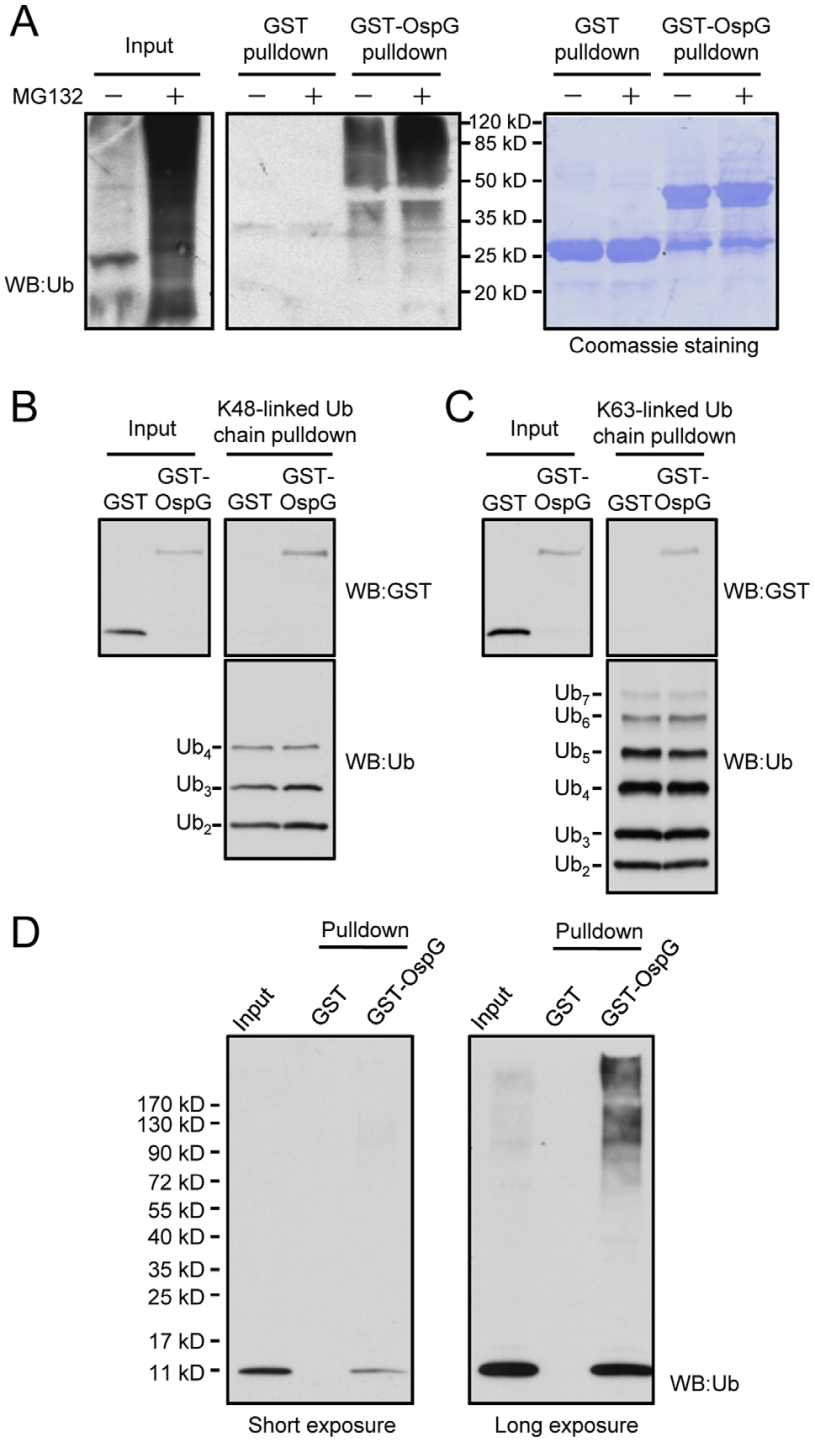
High-affinity binding between OspG and ubiquitin conjugates, poly-ubiquitin chains and free ubiquitin. (**A**) Pulldown of ubiquitin-conjugated proteins by purified GST-OspG. Glutathione-Sepharose beads coated with GST-OspG or GST alone were incubated with lysates of intact 293T cells or MG132- treated 293T cells. Proteins retained on the beads were eluted with SDS loading buffer and separated onto12% SDS-PAGE gels. Shown on the left are anti-ubiquitin immunoblots and on the right are Coomassie blue staining of GST or GST-OspG proteins present on the beads. (**B** and **C**) Pulldown of OpsG by K48- or K63-linked poly-ubiquitin chains. Ni-NTA Sepharose beads coated with His6-ubiquitin chains with indicated linkages were incubated with GST or GST-OspG. Proteins retained on the beads were subjected to SDS-PAGE and anti-GST immunoblotting analysis. (**D**) Pulldown of free ubiquitin by GST-OspG. GST or GST-OspG proteins were immobilized onto Glutathione Sepharose beads and the beads were then incubated with lysates of 293T cells. The interacting proteins eluted from the beads were resolved by 4–20% gradient SDS-PAGE gel and analyzed by anti-ubiquitin immunoblotting.

To search for possible OspG-interacting proteins related to ubiquitination, purified GST-tagged OspG protein (GST-OspG) was immobilized onto glutathione beads and incubated with lysates of intact 293T cells or 293T cells pretreated with the proteasome inhibitor MG132. Proteins retained on the beads were then subjected to reducing SDS-PAGE and anti-ubiquitin (P4D1) Western blotting analysis. Unexpectedly, we found that GST-OspG, but not GST alone, could efficiently pull down a large amount of polyubiquitinated proteins from intact 293T cell lysates, as shown by the heavy smear signals recognized by the anti-ubiquitin antibody (Fig. 1A). When cells were pretreated with MG132 to increase the level of polyubiquitinated proteins, the amounts of polyubiquitinated species precipitated by GST-OspG were also significantly increased and such effects were not observed with pulldown assays performed with GST protein alone (Fig. 1A). These data suggest that OspG can specifically bind to polyubiquitinated proteins in eukaryotic host cells.

As mentioned earlier, polyubiquitin chains with different linkages are associated with different functions. K48-linked ubiquitin chain is established in signaling conjugated substrates for proteasomal degradation while the other prevalent K63 ubiquitin chain regulates the function or activity of the target substrate. To examine whether OspG prefers to bind ubiquitin chains with a particular linkage, His6-tagged ubiquitin chains (Ub2-4) of either K48 or K63 linkage were incubated with GST or GST-OspG. Consistent with the increased precipitation of polyubiquitinated proteins by GST-OspG from MG132-treated cells, GST-OspG, but not GST alone, was found to be efficiently precipitated by K48-linked ubiquitin chains (Fig. 1B). Notably, K63-linked ubiquitin chains (Ub2-7) could also specifically pull down GST-OspG with efficiency comparable to that observed K48 ubiquitin chains (Fig. 1C). These data demonstrate that OspG can directly bind polyubiquitin chains of either K48 or K63 linkage and further indicate that OspG may modulate host ubiquitin signaling that is not limited to proteasomal degradation.

### OspG Directly Binds to Ubiquitin, but not Other Ubiquitin-like Proteins

One interpretation accounting for OspG binding to both K48- and K63-linked ubiquitin chains is that OspG may bind to the monomer form of free ubiquitin, which was missed in the previous cell-lysate pulldown assay because free ubiquitin ran out of the 12% SDS-PAGE gel used in that assay. To this end, samples of GST-OspG pulldown performed with 293T cell lysates were re-examined by Western blotting analysis using 4–20% gradient gel. As shown in Fig. 1D, GST-OspG did precipitate free ubiquitin more efficiently than the polyubiquitin-conjugated proteins. As a control, no binding between ubiquitin and GST alone was observed in this assay.

**Figure 2:**
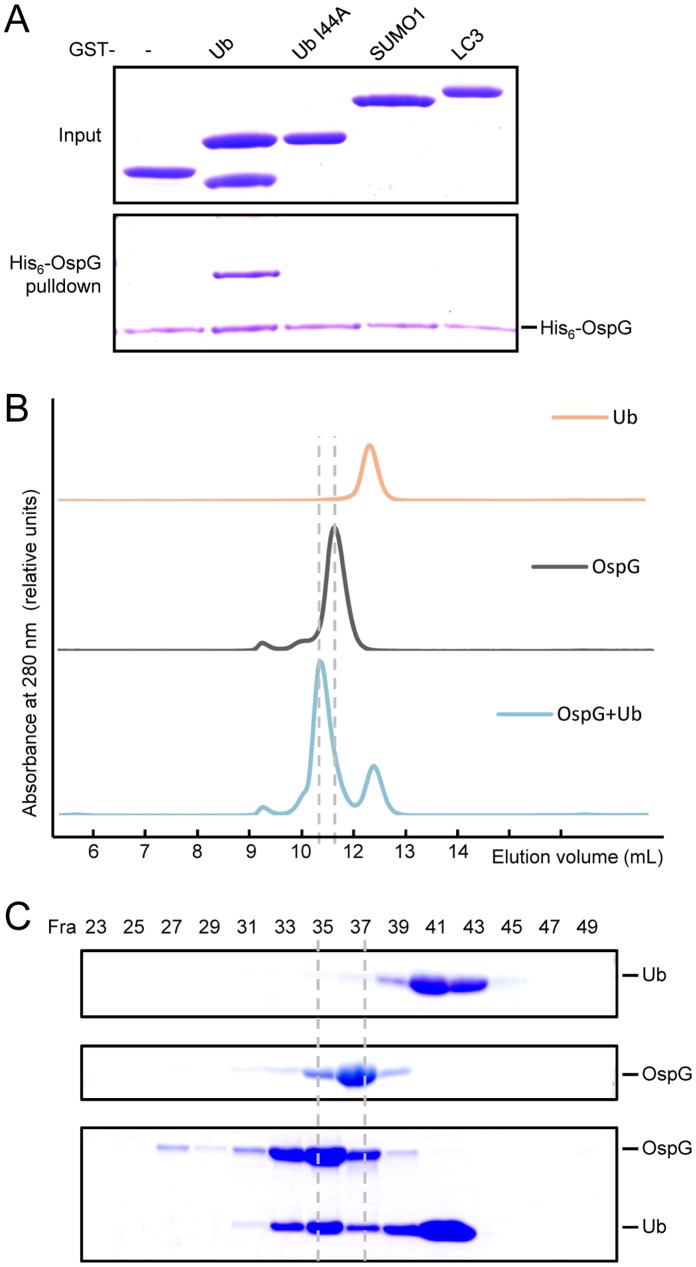
OspG directly and specifically binds to free ubiquitin. (**A**) Pulldown of free ubiquitin by OspG. His6-OspG was immobilized onto Ni-NTA beads and the beads were then incubated with GST, GST-ubiquitin, GST-ubiquitin I44A mutant, GST-LC3 or GST-SUMO1. Bound proteins were eluted from the beads and resolved on the SDS-PAGE gel followed by Coomassie blue staining. (**B** and **C**) Complex formation of ubiquitin and OspG on the gel filtration column. Purified Flag-His6-ubiquitin and His6-OspG proteins were subjected to Superdex-75 gel filtration chromatography individually or after being mixed together. Shown in (B) are the chromatograms. Aliquots of indicated fractions were resolved on the SDS-PAGE gel stained with Coomassie blue in (C).

To confirm the direct interaction between OspG and ubiquitin, purified His6- OspG was used to pull down recombinant GST and GST-ubiquitin. Only GST-ubiquitin was precipitated by His6-OspG and the binding appeared to be highly robust (Fig. 2A). In eukaryotes, there are a large number of ubiquitin-binding modules that confer diverse regulations of ubiquitin signaling. Most of these ubiquitin-binding proteins recognize a critical conserved region on ubiquitin, the I44-centered hydrophobic patch [25], [26]. Interestingly, when I44 of ubiquitin was mutated into Ala, precipitation of this mutant form of ubiquitin by OspG was not observed (Fig. 2A). This result indicates that OspG, similar to most ubiquitin-binding modules in the host, also recognizes the canonical I44 surface of ubiquitin to achieve high-affinity interaction.

Ubiquitin-like proteins (UBL proteins), such as SUMO and LC3, share a three-dimensional structural fold with ubiquitin. These UBLs can be conjugated onto target proteins through a similar enzymatic cascade, which critically regulates many cellular processes, including transcription, DNA repair, signal transduction, autophagy and cell-cycle control [27], [28]. To test whether OspG can interact with other UBLs, His6-OspG was used to pull down GST-SUMO1 and GST-LC3, but no interaction was observed. This result suggests that OspG selectively binds to ubiquitin among the UBL-family members (Fig. 2A).

The interaction between OspG and ubiquitin was further tested by elution on a gel filtration column. Purified OspG (23.6 kDa) and ubiquitin (12.1 kDa), when loaded onto the column separately, were eluted at the volume corresponding to the molecular weight of their monomeric forms (Fig. 2B). When OspG was mixed with excess ubiquitin and the mixtures were loaded onto the same column, a complex species eluted earlier than OspG and ubiquitin alone appeared (Fig. 2B). The ∼ 10.5 ml elution volume of this species corresponds to roughly ∼ 50 kDa for a globular protein, suggesting a possible 1∶1 stoichiometry for OspG and ubiquitin binding. Further SDS-PAGE analysis of each fraction confirmed the co-elution of OspG and ubiquitin as well as the roughly estimated 1∶1 OspG-ubiquitin complex (Fig. 2C).

### Both the Carboxyl Terminus and the Kinase-like Domain of OspG are Required for Ubiquitin Binding

**Figure 3:**
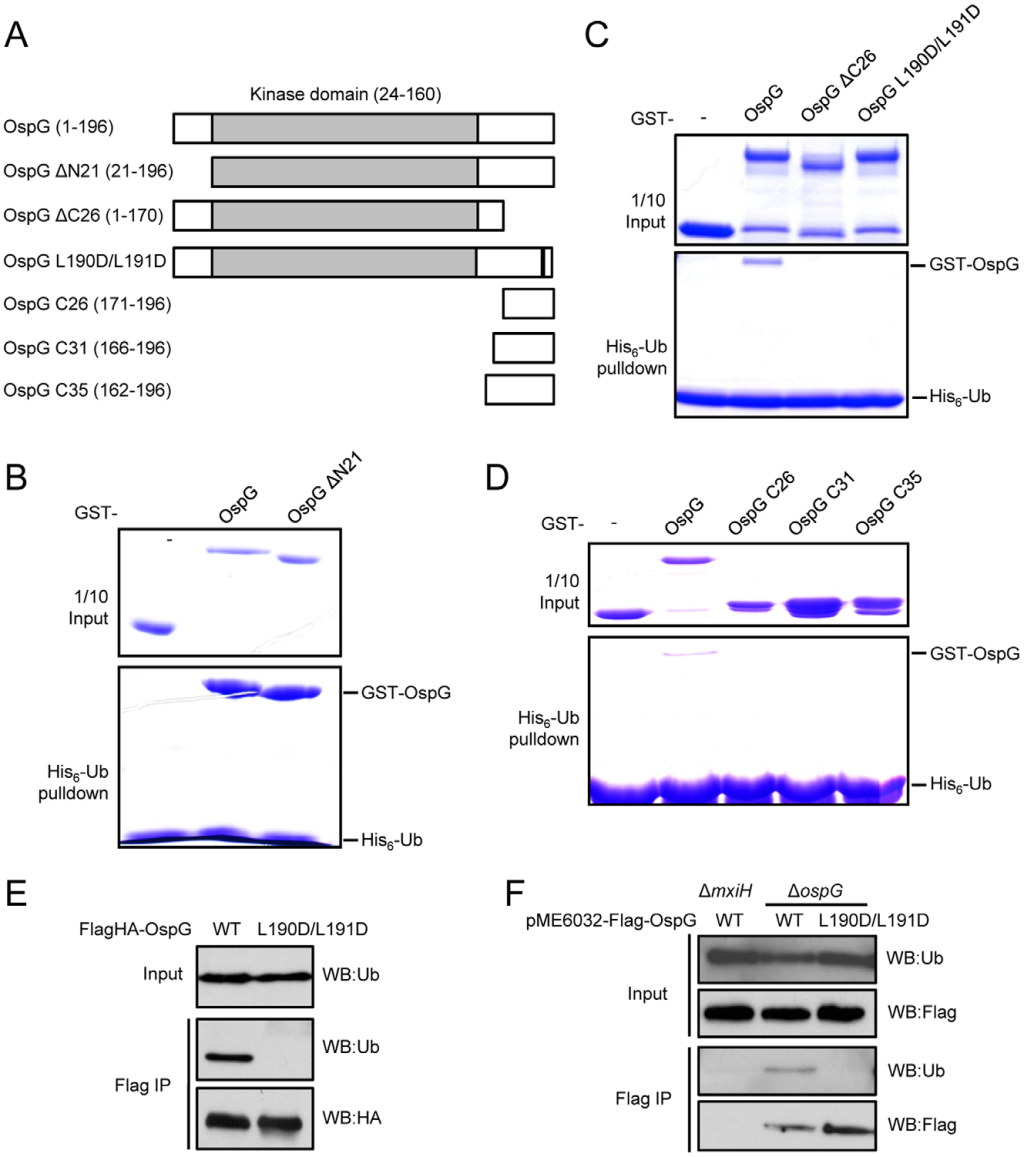
The carboxyl terminus of OspG is required for interaction with ubiquitin. (**A**) Schematic presentation of OspG truncations assayed in (B-D). The gray box donates the region that shows sequence similarity to eukaryotic serine/threonine kinase sub-domains I-VII. (**B**, **C** and **D**) Pulldown assays of the binding between ubiquitin and various OspG truncation proteins. Ni-NTA Sepharose beads coated with His6-ubiquitin were incubated with GST, GST-OspG or indicated GST-OspG truncation proteins. Proteins retained on the beads were eluted and then subjected to SDS-PAGE and Coomassie blue staining analysis. (**E**) Coimmunoprecipitation assay of OspG (WT or L190D/L191D mutant) and ubiquitin interaction in transfected HEK 293T cells. Shown are immunoblots of anti-Flag immunoprecipitates (Flag IP) and total cell lysates (Input). (**F**) Assay of OspG and ubiquitin interaction during *Shigella* infection. HEK 293T cells were infected with indicated *Shigella* deletion/complementation strains. Lysates of infected cell were subjected to anti-Flag immunoprecipitation. Δ*mxiH* is a type III secretion deficient strain; pME6032 is a rescue plasmid for expressing Flag-OspG (wild-type or L190D/L191D mutant) in the bacteria. Shown are immunoblots of anti-Flag immunoprecipitates (Flag IP) and total cell lysates (Input).

**Figure 4:**
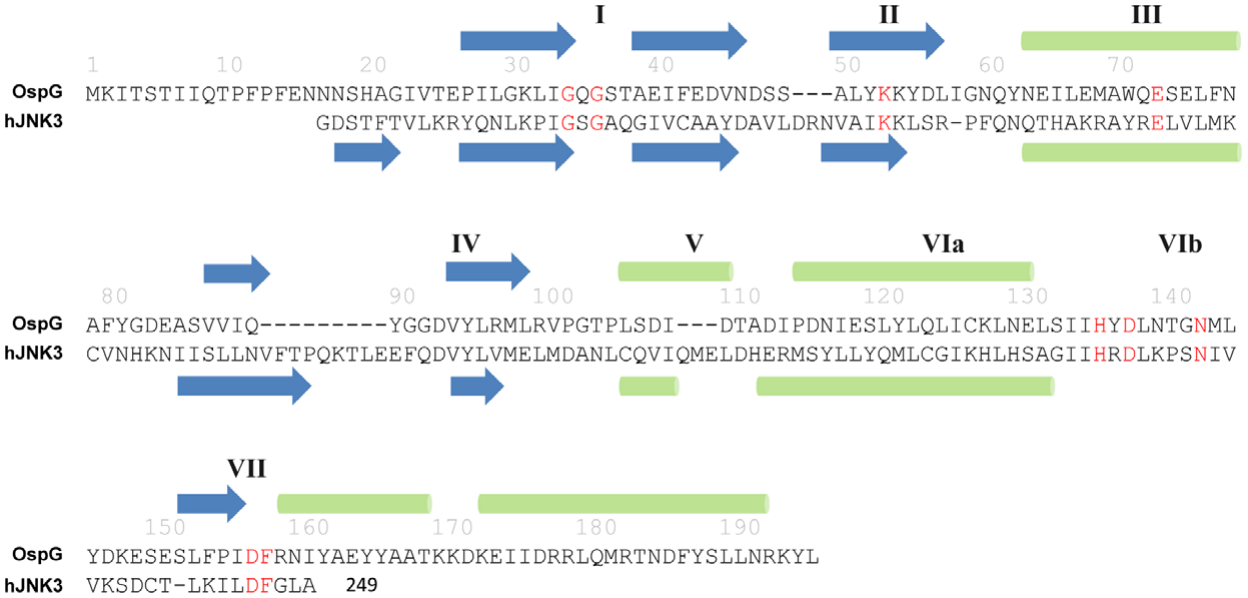
Secondary structure-based sequence alignment of OspG with human JNK3. The protein names are indicated at the left of the alignment. The key residues in the kinase domain are colored in red. Predicted secondary structures of OspG are shown on top of the OspG sequence. Secondary structures determined from crystal structure of JNK3 are shown underneath the sequence of JNK3 in the alignment. Green ovals are α helices and blue arrows are β strands. The sub-domains are labeled with Roman numerals.

OspG is a small-size protein with only 196 amino acids and its sequence from residue 24 to 160 exhibits confident similarities to sub-domains I to VII of eukaryotic serine/threonine kinase (Fig. 3A and Fig. 4). The extreme carboxyl terminus (residues 161–196), predicted to form an α helix, does not show any sequence similarity to known proteins. The fact that no classical ubiquitin-binding domains or motifs could be identified in OspG indicates a novel ubiquitin-binding mode and also promoted us to further investigate its ubiquitin binding mechanism. Truncation of the amino terminal 21 residues preceding the putative kinase domain did not affect OspG binding to ubiquitin in the *in vitro* pulldown assay (Fig. 3B). Notably, removal of the extreme carboxyl terminal 26 residues in OspG completely abolished its interaction with ubiquitin (Fig. 3C), suggesting that the carboxyl terminal region following the kinase-like domain in OspG is essential for ubiquitin binding. Mutation of two leucine residues (L190D/L191D) in this region abolishedOspG binding to ubiquitin (Fig. 3C), suggesting the importance of these two residues. Furthermore, when the carboxyl terminal 26, 31, or 35 residues were artificially fused to GST protein and the fusion proteins were assayed for co-precipitation by ubiquitin, none of the three chimeric proteins were capable of binding to ubiquitin (Fig. 3D). These data suggest that the carboxyl terminal helical region in OspG is essential but not sufficient for mediating specific interaction with ubiquitin and the kinase-like domain is also involved in ubiquitin binding.

We also tested OspG and ubiquitin interaction in eukaryotic host cells. Transient expression of OspG into 293T cells could efficiently co-immunoprecipitate endogenous ubiquitin (Fig. 3E). In contrast, mutation of the carboxyl-terminal two leucine residues (L190D/L191D) in OspG abolished the interaction in this transfection assay (Fig. 3E). To confirm this interaction during *Shigella* infection, an *ospG* deletion strain of *S. flexneri* was constructed. A Flag-tagged OspG was transformed into the deletion strain and infection was then performed. As shown in Fig. 3F, Flag-OspG expressed in the bacteria could co-immunoprecipitate endogenous ubiquitin from infected 293T cells, and importantly the L190D/L191D mutant was also defective in interacting with ubiquitin in this infection assay. As another control, OspG expressed in the type III secretion deficient Δ*mxiH* strain did not co-immunoprecipitate ubiquitin from infected 293T cells (Fig. 3F). These data demonstrate the type III-delivered OspG can bind to host ubiquitin and the interaction also requires the carboxyl terminal helical region in OspG.

### OspG and OspG-like Effectors are a Type of Atypical Serine/threonine Kinases

OspG shows sequence similarity to eukaryotic protein serine/threonine kinase [24]. A typical serine/threonine kinase domain can be divided into eleven sub-domains (I-VI, VIIA, VIIB, VIII-XI) [29], [30]. Aligning the sequence of OspG to the kinase domain of a prototypical eukaryotic serine/threonine kinase JNK3 shows that residues from 24 to 160 of OspG are homologous to JNK3 kinase sub-domains I-VII (Fig. 4). The predicted secondary structures of OspG can also be aligned well to those of JNK3 sub-domains I-VII, and all the catalytically important residues within these domains are maintained in OspG (Fig. 4). However, OspG apparently lacks the kinase sub-domains VIII–XI (Fig. 4). The sub-domain VIII is the activation loop whose conformational change usually stimulates the kinase activity upon serine/threonine phosphorylation of this loop by an upstream kinase or itself [29]. The sub-domains IX and X are generally required for maintaining the overall structural integrity [29]. Thus, OspG appears to be a half kinase with all necessary catalytic residues.

**Figure 5:**
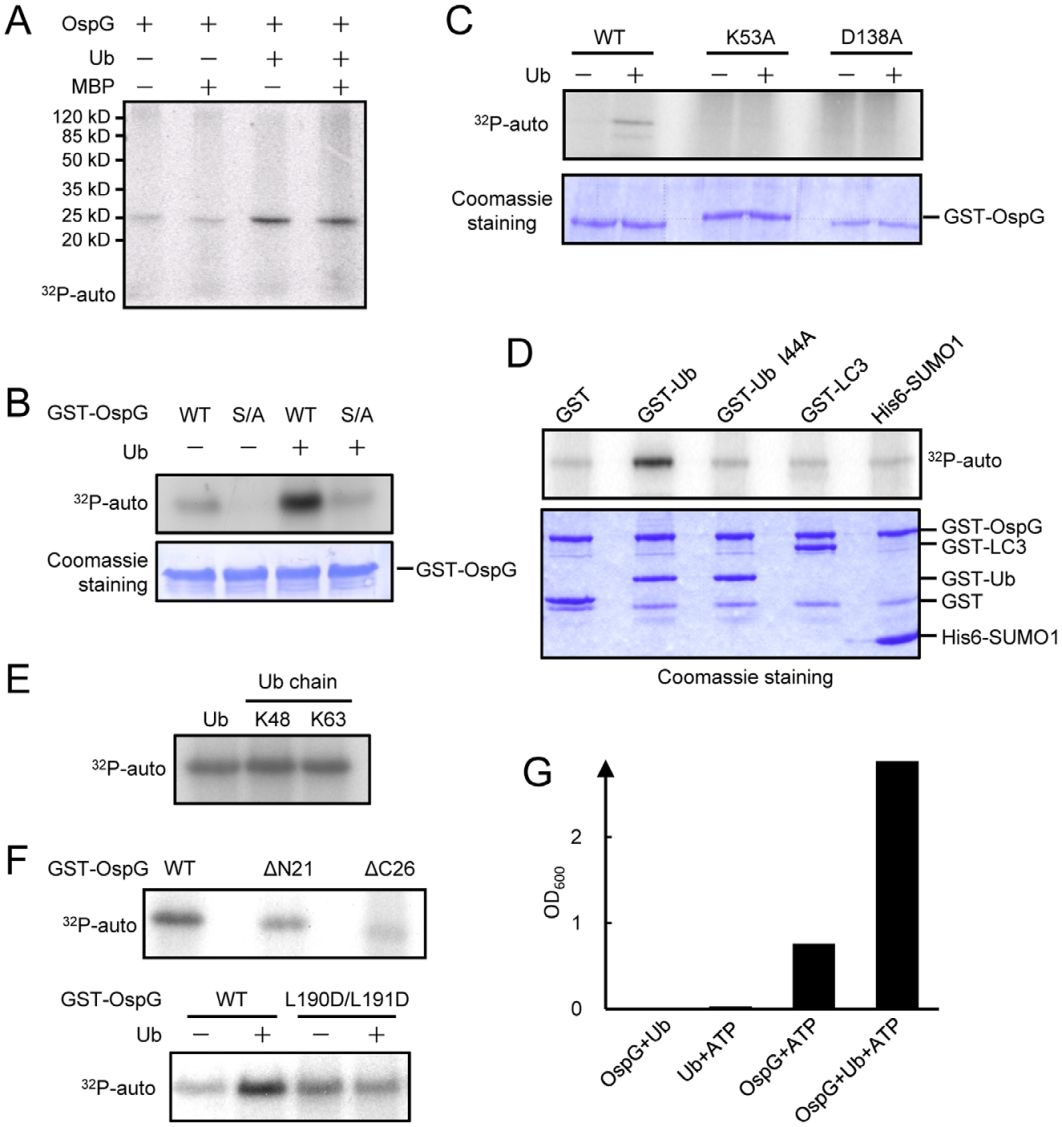
Ubiquitin binding stimulates the autophosphorylation and ATP hydrolysis activity of OspG. (**A**) Kinase assay of OspG in the presence or absence of ubiquitin. Purified OspG protein was incubated with [γ-P^32^] ATP with or without the addition of ubiquitin into the reaction. Myelin basic protein (MBP) was included as the possible artificial substrate. Incorporation of phosphate into the protein was examined by autoradiography of the reaction mixtures resolved on the SDS-PAGE gel as shown. (**B**) Effects of mutation in the autophosphorylation site of GST-OspG. The autophosphorylated serine residue in the linker region of GST-OspG was mutated into alanine and the mutant protein (S/A) was subjected to the kinase assay in the presence or absence of ubiquitin as described in (A). (**C**) Effects of mutations in the putative catalytic residues on ubiquitin stimulation of OspG kinase activity. Wild-type or indicated mutant GST-OspG (K53A and D138A) proteins were used for the autophosphorylation assay in the presence or absence of ubiquitin. Autophosphorylation of OspG is shown by autoradiography in the upper panel. Coomassie blue staining of GST-OspG proteins are shown in the lower panel. (**D**) Autophosphorylation assay of GST-OspG in the presence of GST-tagged ubiquitin, ubiquitin I44A, LC3 or SUMO1. (**E**) Autophosphorylation assay of GST-OspG in the presence of free ubiquitin, K48- or K63-linked poly-ubiquitin chains. (**F**) Autophosphorylation assay of GST-OspG, GST-OspG ΔN23, GST-OspG ΔC26 or GST-OspG L190D/L191D mutant. The assays were all performed in the presence of ubiquitin. (**G**) ATP hydrolysis assay of OspG and effects of addition of ubiquitin. His6-OspG and ubiquitin were incubated together with or without ATP supplementation. Release of the phosphate following the reaction was detected by measuring the phospho-molybdate complex formation on a spectrometer (absorbance at 600 nm).

Given that no physiological substrates of OspG have been reported, we performed the kinase assay by using a generic artificial substrate myelin basic protein (MBP) and examined both MBP phosphorylation and OspG autophosphorylation either in the absence or presence of ubiquitin. While no MBP phosphorylation was detected, phosphorylation of GST-OspG by itself was observed (Fig. 5A), which confirms the observation made in the previous study [24]. Given that the autophosphorylation signal is rather weak, we further purified MBP-OspG and His6-OspG and assayed their autophosphorylation activity. Interestingly, no autophosphorylation occurred on MBP-OspG and His6-OspG (data not shown). Through mass spectrometry analysis, the autophosphorylation site of GST-OspG was mapped to a serine residue located in the linker region between the GST and OspG (Fig. S1A). This observation not only explains why no “autophosphorylation” occurred on MBP-OspG and His6-OspG, but also indicates that the observed “autophosphorylation” of GST-OspG differs from the classical kinase autophosphorylation that usually occurs on the activation loop. To confirm the kinase activity of OspG, we then assayed two other commercially available artificial substrates, casein from bovine milk and histone from calf thymus. While no phosphorylation of casein was observed, an evident phosphorylation signal was detected in the crude histone extract (Fig. S1B). Taken together, these data provide definitive experimental evidences for the kinase activity of recombinant OspG protein.

OspG has homologous effector proteins in other bacterial pathogens, including NleH1/2 in EPEC and enterohaemorrhagic *E. coli* (EHEC), and NleH in the mouse pathogen *Citrobacter rodentium*, which are all closely related diarrheagenic pathogens. OspG and NleHs share evident sequence similarities within the kinase domain (also sub-domains I-VII only) (Fig. S2A). In addition, NleH1/2 bears an extended N-terminal region that lacks the similarity to OspG. GST-NleH1 and GST-NleH2 could also phosphorylate themselves (Fig. S2B), similarly to that observed with GST-OspG. However, when the catalytic lysine residue important for ATP binding was mutated to alanine, no autophosphorylation of GST-NleH1/2 could be detected [31] (Fig. S2B). These results confirm that OspG and OspG-like bacterial effectors harbor serine/threonine kinase activity *in vitro*.

### Ubiquitin Binding Stimulates the Kinase Activity of OspG

OspG binds to free ubiquitin, poly-ubiquitin chains and even ubiquitin-conjugated proteins. It is intuitively difficult to imagine that the function of OspG is to target host ubiquitin signaling as we hypothesized originally. Given that several type III effectors require binding to abundant host factors for their own activation, we then hypothesize that OspG may use ubiquitin binding to modulate its own biochemical activity, i.e. the kinase activity. Most notably, addition of ubiquitin into the phosphorylation reaction drastically promoted GST-OspG autophosphorylation (Fig. 5A) as well as phosphorylation of the crude histone extract (Fig. S1B). This ubiquitin-stimulated auto-phosphorylation was completely abolished when the serine residue in the linker region was mutated into alanine (Fig. 5B), suggesting a similar nature of autophosphorylation upon ubiquitin stimulation. Lys-53 of OspG is the predicted catalytic residue that anchors and orients the ATP, and Asp-138 is the potential catalytic residue to accept the proton from the attacking hydroxyl group. When these two residues were individually mutated toAla, autophosphorylation of GST-OspG was undetectable even in the presence of ubiquitin (Fig. 5C). This excludes the possibility that phosphorylation of GST-OspG comes from a contamination activity. In contrast to wild-type ubiquitin, ubiquitin I44A mutant or other UBL proteins did not stimulate the autophosphorylation of GST-OspG (Fig. 5D), which is consistent with their deficient binding to OspG. Similarly to ubiquitin, both K48-linked and K63-linked ubiquitin chains also promoted the autophosphorylation of GST-OspG (Fig. 5E). Deletion of the carboxyl terminal 26 residues from OspG that resulted in a defective ubiquitin binding severely attenuated the phosphorylation of GST-OspG (Fig. 5F). Similarly, autophosphorylation of GST-OspG L190D/L191D double mutant was not responsive to ubiquitin stimulation (Fig. 5F). Moreover, ubiquitin addition did not promote the autophosphorylation of GST-NleH1/2 (Fig. S2B), which is consistent with their lack of a carboxyl terminal sequence required for ubiquitin binding in OspG (Fig. S2A). In all above kinase assays, phosphorylation of ubiquitin was never observed (Fig. 5A). These data strongly support the hypothesis that ubiquitin is not a target of OspG but instead regulates the kinase activity of OspG.

Some kinases have been demonstrated to have intrinsic ATPase activity [32], [33], [34], [35]. When His6-OspG was incubated with ATP and the phosphate release was detected by molybdate dye/phosphate complex formation, OspG exhibited intrinsic ATPase activity, which was also significantly increased by addition of ubiquitin (Fig. 5G). As expected, ubiquitin alone did not promote phosphate release from ATP. This again confirms our hypothesis that ubiquitin binding functions to stimulate the biochemical activity of OspG when it is translocated into host cells.

### Ubiquitin Binding is Required for the Function of OspG in Attenuating Host NF-κB Signaling during Infection

**Figure 6:**
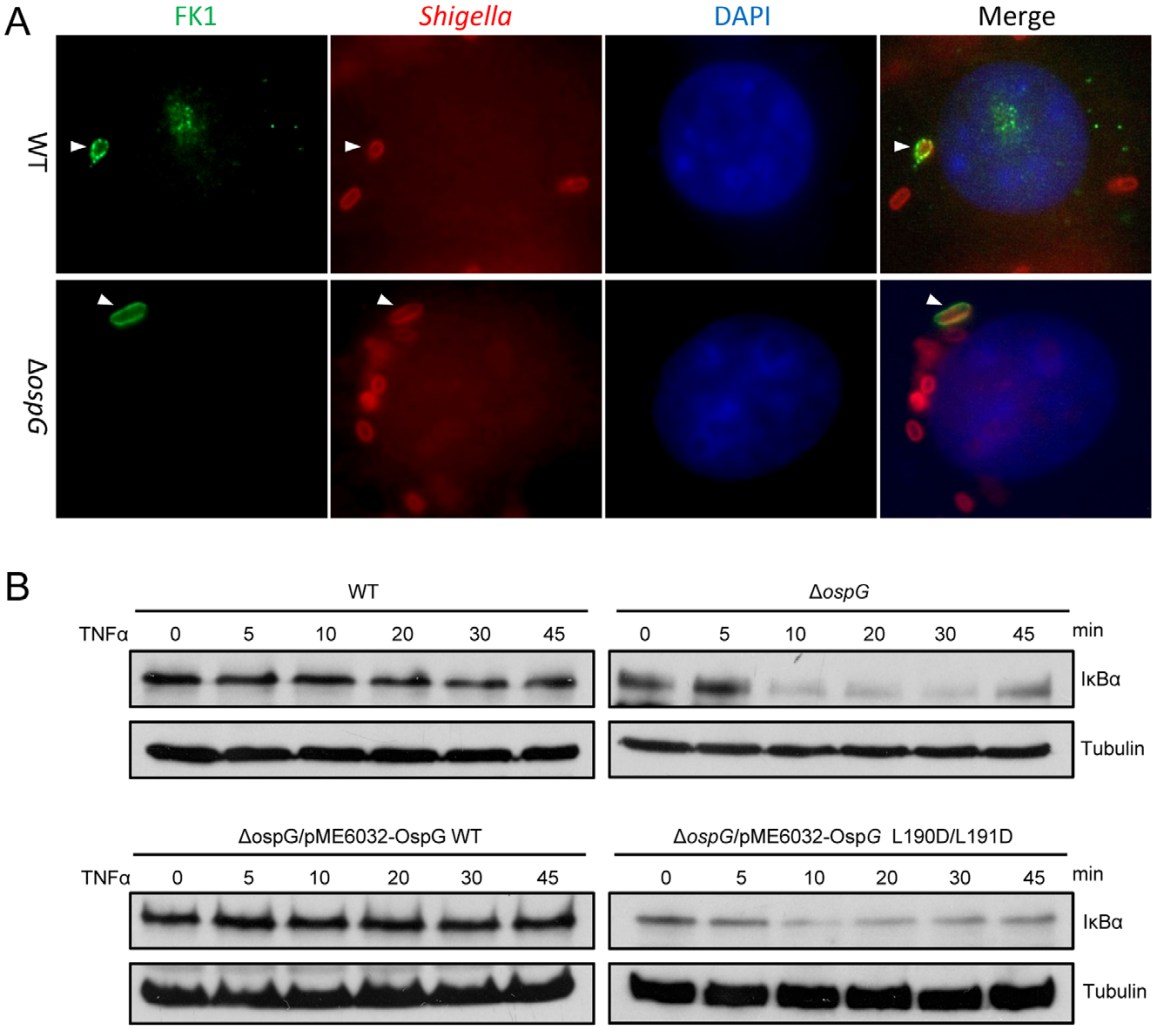
Ubiquitin binding of OspG is required for inhibiting host NF-κB signaling during *Shigella* infection. (**A**) The role of OspG in recruitment of ubiquitin to intracellular *Shigella*. HeLa cells were infected with wild type (WT) or Δ*ospG* mutant *S. flexneri* strain. Polyubiquitinated proteins stained by the FK1 antibody (green), intracellular bacteria stained by anti-*Shigella* antibody (red) and DAPI-stained nuclei (blue) were visualized by fluorescence microscopy. (**B**) The role of OspG ubiquitin binding activity in inhibiting NF-κB activation in *Shigella*-infected cells. HeLa cells were infected with indicated *S. flexneri* strains. Infected cells were treated with TNFα to stimulate NF-κB pathway activation. Lysates of infected cell collected at indicated time points after the stimulation were subjected to anti-IκBα and anti-tubulin immunoblotting analyses.

Ubiquitin or ubiquitin-chain decoration of intracellular bacteria has been proposed as a signal that triggers autophagy-mediated host defense. To probe the functional consequence of OspG ubiquitin binding and activation of its kinase activity during *Shigella* infection, we first checked whether OspG plays a role in ubiquitin-chain formation surrounding intracellular *Shigella*. Using an antibody specific for polyubiquitinated proteins (FK1), we were able to detect some polyubiquitin signals around a portion of intracellular *S. flexneri* in infected HeLa cells, but this effect was not affected by deletion of *ospG* from the bacteria (Fig. 6A). This suggests that OspG does not play a role in recruitment or generation of polyubiquitinated proteins around *S. flexneri* during infection. Previous study suggests that OspG may interfere with the innate immune NF-κB signaling in *S. flexneri*-infected host cells [24]. Confirming this observation, TNFα treatment induced a more rapid degradation of IκBα inhibitory protein in HeLa cells infected with the Δ*ospG* strain compared with that in wild-type strain (Fig. 6B). While re-expression of wild-type OspG in the deletion strain restored the inhibition of TNFα-induced IκBα degradation, the ubiquitin binding-deficient L190D/L191D mutant of OspG failed to complement the deletion strain in blocking IκBα degradation (Fig. 6B). These results suggest that OspG requires binding to host ubiquitin to exert its function in suppressing host innate immune signaling, which is likely through activation of its kinase activity.

## Discussion

In summary, we have discovered that *Shigella* type III effector OspG directly and specifically interacts with host ubiquitin through the I44 hydrophobic patch on the ubiquitin surface. OspG also binds to poly-ubiquitin chains and ubiquitin-conjugated proteins. Binding to ubiquitin stimulates the kinase and intrinsic ATP hydrolysis activity of OspG, which, as an atypical kinase, exhibits sequence similarity to eukaryotic serine/threonine kinase sub-domains I-VII. Our data suggest a novel mechanism of activating a bacterial virulence effector by the host ubiquitin system. Our data also demonstrate that OspG and OspG-like effectors, NleHs from EPEC/EHEC, are atypical kinases and do exhibit *in vitro* kinase activity. It is important for future studies to identify the host target or kinase substrate of OspG, which shall advance our understanding of OspG binding to ubiquitin as well as its functional role in *Shigella* pathogenesis. *Shigella* secrets the IpaH family of ubiquitin ligase effectors and the OspI effector that targets host Ubc13 for deamidation. Therefore, ubiquitin binding and activation of OspG provides an additional layer of complexity to the interplay between *Shigella* and the eukaryotic ubiquitin system, regardless of whether the substrate of OspG kinase is related to the ubiquitin system or not.

The activity of bacterial virulence effectors is often under tight and sophisticated regulation, through which the bacteria can manipulate host processes efficiently and effectively. Turning on the biochemical activity of bacterial type III effectors by binding to host factors have been shown for several other effectors in recent studies. The canonical *Yersinia* serine/threonine kinase effector YpkA has a low or undetectable kinase activity in the bacteria, which is significantly stimulated by actin binding to its extreme carboxyl terminal region [36]. Another type III effector AvrRpt2 from plant pathogen *Pseudomonas syringae* binds to plant cyclophilin protein to turn on its cysteine protease activity [37]. An extensively studied *Yersinia* type III effector YopJ harbors an O-acetyltransferase activity that is promoted by host small molecule inositol hexakisphosphate (IP6) [38]. All these effectors appear to bind to a rather abundant host factor for activation, and this feature also applies to OspG as ubiquitin is one of the most abundant proteins in eukaryotic cells. A general assumption for host activation of bacterial virulence factors is that bacteria, in this way, can prevent effector activities that might be harmful to themselves. This could be the case for the three characterized effectors mentioned above. However, due to the fact that OspG lacks the kinase activation loop as well as the kinase sub-domains VIII–XI, it is also possible that requiring of binding to a host factor for OspG activation might result from the intrinsic structural property of OspG and OspG-like atypical kinase effectors. This further predicts the presence of other distinct host factors that are required for activation of OspG-like NleHs effectors from EPEC/EHEC.

## Materials and Methods

### Plasmids and Reagents

OspG gene was PCR amplified from the *Shigella flexneri* 2a strain 301. For recombinant expression in *E. coli*, OspG was constructed into pGEX-6P-2 and pET21a vectors. cDNAs for ubiquitin, SUMO1 and LC3 were also subcloned into pGEX-6p-2 to generate GST fusion proteins. Point mutations were generated by the QuikChange site-directed mutagenesis kit (Stratagene). All of the plasmids were verified by DNA sequencing. His6-tagged poly-ubiquitin chains (K48-linked and K63-linked) were purchased from Boston Biochem. Anti-GST (sc-138) and anti-ubiquitin (P4D1, sc-8017) antibodies were from Santa Cruz Biotechnology. Artificial kinase substrates casein from bovine milk and histone from calf thymus were both purchased from Sigma.

### Protein Expression and Purification

All recombinant proteins were expressed and purified from *E. coli* BL21 (DE3) strain. Protein expression was induced at room temperature (RT) with 0.4 mM isopropyl-b-D-thiogalactopyranoside (IPTG) for 12 hours when OD_600_ reached 0.6. Cells were harvested and re-suspended in the lysis buffer containing 25 mM HEPES (pH 7.4) and 150 mM NaCl. Cells were lysed by sonication, and the debris was pelleted at 16,000 rpm for 60 minutes. The supernatant was then applied to the corresponding resins for affinity purification. The GST fusion protein was purified by using glutathione-Sepharose resin (GE Healthcare). The His6-tagged fusion protein was purified by using Ni-NTA resin (Qiagen).The resins were washed with the lysis buffer and then eluted with glutathione or immidazole-containing buffer. Eluted proteins were dialyzed against the lysis buffer, concentrated and stored in 20% glycerol at −20°C. Concentrations of purified recombinant proteins were determined by Coomassie blue staining of SDS-PAGE gels using the BSA standards.

### Cell Culture and Immunoprecipitation

HEK 293T cells obtained from ATCC were grown in DMEM (Hyclone) supplemented with 10% fetal bovine serum (Gibco) and 2 mM L-glutamine (Hyclone) at 37°C in a 5% CO_2_ incubator. To prepare the cell extracts, approximately 1×10^7^ cells were washed once with PBS and re-suspended in 1 ml of the lysis buffer (25 mM HEPES, pH 7.4,150 mM NaCl,1 mM DTT, and 1% Triton X-100). The cell lysates were placed on ice for 15 minute and then spun at 13,000 rpm for 15 minute to collect the supernatant. To block proteasomal degradation, cells were treated with 5 µM MG132 for 3 hours prior to lysis. For immunoprecipitation, 293T cells were transiently transfected with Flag-HA-OspG (wild type or indicated mutants). After 24 hours, cells were washed with PBS and lysed for 15 min in a buffer containing 50 mM HEPES (pH 7.4), 150 mM NaCl, and 1% Triton X-100.The lysates were cleared by centrifugation and proteins were immunoprecipitated with anti-Flag M2 beads (Sigma). The immunoprecipitates were washed four times with the lysis buffer and subjected to immunoblotting analysis.

### Pulldown and Analytical gel-filtration Binding Assays

For the pulldown assay, ∼ 2 µg of proteins were immobilized on Ni-NTA or glutathione-Sepharose resins and the resins were then incubated with 20 µg of prey proteins in 1 mL of binding buffer (25 mM HEPES, pH7.4, 150 mM NaCl, 1% Triton X-100) at 4°C for one hour. The resins were then centrifuged and extensively washed with the binding buffer. Proteins retained on the resins were eluted with an SDS loading buffer (50 mM Tris-HCl, pH6.8, 2% SDS, 10% glycerol, and 100 mM DTT).

To analyze complex formation on the gel filtration column, 200 µg of His6-OspG (23.6 kDa) and 100 µg of Flag-His6-ubiquitin (12.1 kDa) proteins were loaded onto the 24-ml Superdex 75 10/300 column (GE Healthcare) individually or after they were pre-mixed. The column buffer for chromatography contains 25 mM HEPES, pH 7.4, 150 mM NaCl and 1 mM DTT. Fractions of 300 µl each were collected. Aliquots of every other fraction were subjected to SDS-PAGE and Coomassie blue staining analysis.

### Kinase and ATP Hydrolysis Assays

2 µg of OspG or GST-OspG were incubated with 2 µCi of [γ-P^32^] ATP in the kinase assay buffer containing 10 mM HEPES, pH 7.4,10 mM MgCl_2_ and 10 mM cold ATP at 30°C for 30 minutes.1 µg of ubiquitin and myelin basic protein (MBP) were added into the reaction as indicated. The reactions were quenched with the SDS loading buffer, loaded onto a 15% SDS-PAGE gel and transferred to a PVDF membrane (Millipore). Radioactivity was analyzed by autoradiography.

The ATPase activity of His6-OspG was determined by measuring the release of the inorganic phosphate. The reaction was carried out at 30°C for 30 minutes in a buffer containing 25 mM HEPES (pH 7.4), 150 mM NaCl, 10 mM MgCl_2_, and 0.5 mM ATP. The reaction was initiated by addition of purified OspG, and quenched by adding 50 µl of molybdate dye/additive mixture (Promega). Following color development, the released inorganic phosphates were measured at the absorbance of 600 nm.

### Genetic Manipulation of *Shigella* and Infection Assays

In-frame deletion of *ospG* from *Shigella flexneri* 2a 2457T was performed as previously described [11]. For infection, cultures of indicated *Shigella* strains were added to HeLa cells at MOI of 100 followed by centrifugation at 800 g for 10 min at RT to facilitate bacterial attachment. Infection was performed at 37°C for one hour, and the cells were then washed with PBS and incubated for additional four hours in fresh culture medium supplemented 100 ug/ml gentamicin to kill the extracellular bacteria. To examine ubiquitin recruitment to the bacteria, infected cells were fixed and subjected to immunofluorescence analysis. Bacteria were stained with anti-*Shigella* antibody (Abcam) and the polyubiquitinated proteins were stained with FK1 antibody (Millipore). To assay IκBα degradation, infected cells were treated with 10 ng/mL TNFα for indicated time periods. The cells were then lysed in 2X Laemmli buffer, and the protein samples were loaded onto an SDS-PAGE gel for immunoblotting analysis. For complementation in Δ*ospG* strain, 1 mM IPTG was added to the culture medium to induce OspG expression. To test OspG-ubiquitin interaction, HEK 293T cells were infected with indicated *Shigella* strains harboring Flag-OspG expression plasmid (the Flag tag was inserted into OspG after Asn-17) at MOI of 100 for five hours. Cells were lysed in a buffer containing 25 mM HEPES (pH 7.4), 150 mM NaCl and 0.1% Triton X-100. Cell lysates were cleared by centrifugation and then subjected to anti-Flag immunoprecipitation.

## Supporting Information

Figure S1 **Autophosphorylation site in GST-OspG and kinase assay of OspG.** (**A**) Shown is the amino acid sequence of recombinant GST-OspG protein used in the kinase assay and subjected to mass spectrometry analysis. Residues in red correspond to the tryptic peptide sequence identified by mass spectrometry analysis. The sequence of OspG is underlined. The serine residue identified as the phosphorylation site is shown on a blue background. (**B**) OspG kinase assay using commercial histones as the artificial substrates. The assay was performed in the absence or presence of ubiquitin.
(TIF)

Figure S2 **Ubiquitin does not stimulate the kinase activity of OspG-like effector NleH1/2.** (**A**) Sequence alignment of *Shigella flexneri* OspG with NleH1/2 from enteropathogenic *Escherichia coli* O55:H7 (strain CB9615). The protein names are listed on the left of the alignment. Conserved residues are marked in pink, and similar residues are shown in green. (**B**) The kinase assay of GST-NleH1 (WT and K159A mutant) and GST-NleH2 (WT and K169A mutant) in the absence or presence of ubiquitin.
(TIF)

## References

[1] Sansonetti PJ (1998) Pathogenesis of shigellosis: from molecular and cellular biology of epithelial cell invasion to tissue inflammation and vaccine development. Jpn J Med Sci Biol 51 Suppl: S69–80 10.7883/yoken1952.51.supplement1_s69 10211438

[2] Sasakawa C, Buysse JM, Watanabe H (1992) The large virulence plasmid of Shigella. Curr Top Microbiol Immunol 180: 21–44. 1505205 10.1007/978-3-642-77238-2_2

[3] Phalipon A, Sansonetti PJ (2007) Shigella’s ways of manipulating the host intestinal innate and adaptive immune system: a tool box for survival? Immunol Cell Biol 85: 119–129. 17213832 10.1038/sj.icb7100025

[4] Ogawa M, Handa Y, Ashida H, Suzuki M, Sasakawa C (2008) The versatility of Shigella effectors. Nat Rev Microbiol 6: 11–16. 18059288 10.1038/nrmicro1814

[5] Li H, Xu H, Zhou Y, Zhang J, Long C, et al (2007) The phosphothreonine lyase activity of a bacterial type III effector family. Science 315: 1000–1003. 17303758 10.1126/science.1138960

[6] Arbibe L, Kim DW, Batsche E, Pedron T, Mateescu B, et al (2007) An injected bacterial effector targets chromatin access for transcription factor NF-kappaB to alter transcription of host genes involved in immune responses. Nat Immunol 8: 47–56. 17159983 10.1038/ni1423

[7] Kramer RW, Slagowski NL, Eze NA, Giddings KS, Morrison MF, et al (2007) Yeast functional genomic screens lead to identification of a role for a bacterial effector in innate immunity regulation. PLoS Pathog 3: e21. 17305427 10.1371/journal.ppat.0030021 PMC1797620

[8] Huang Z, Sutton SE, Wallenfang AJ, Orchard RC, Wu X, et al (2009) Structural insights into host GTPase isoform selection by a family of bacterial GEF mimics. Nat Struct Mol Biol 16: 853–860. 19620963 10.1038/nsmb.1647 PMC5130228

[9] Ohya K, Handa Y, Ogawa M, Suzuki M, Sasakawa C (2005) IpgB1 is a novel Shigella effector protein involved in bacterial invasion of host cells. Its activity to promote membrane ruffling via Rac1 and Cdc42 activation. J Biol Chem 280: 24022–24034. 15849186 10.1074/jbc.M502509200

[10] Handa Y, Suzuki M, Ohya K, Iwai H, Ishijima N, et al (2007) Shigella IpgB1 promotes bacterial entry through the ELMO-Dock180 machinery. Nat Cell Biol 9: 121–128. 17173036 10.1038/ncb1526

[11] Dong N, Zhu Y, Lu Q, Hu L, Zheng Y, et al (2012) Structurally Distinct Bacterial TBC-like GAPs Link Arf GTPase to Rab1 Inactivation to Counteract Host Defenses. Cell 150: 1029–1041. 22939626 10.1016/j.cell.2012.06.050

[12] Schwartz AL, Ciechanover A (1999) The ubiquitin-proteasome pathway and pathogenesis of human diseases. Annu Rev Med 50: 57–74. 10073263 10.1146/annurev.med.50.1.57

[13] Komander D, Rape M (2012) The ubiquitin code. Annu Rev Biochem 81: 203–229. 22524316 10.1146/annurev-biochem-060310-170328

[14] Rytkonen A, Holden DW (2007) Bacterial interference of ubiquitination and deubiquitination. Cell Host Microbe 1: 13–22. 18005678 10.1016/j.chom.2007.02.003 PMC7173291

[15] Jiang X, Chen ZJ (2012) The role of ubiquitylation in immune defence and pathogen evasion. Nat Rev Immunol 12: 35–48. 10.1038/nri3111 PMC3864900 22158412

[16] Cui J, Yao Q, Li S, Ding X, Lu Q, et al (2010) Glutamine deamidation and dysfunction of ubiquitin/NEDD8 induced by a bacterial effector family. Science 329: 1215–1218. 20688984 10.1126/science.1193844 PMC3031172

[17] Sanada T, Kim M, Mimuro H, Suzuki M, Ogawa M, et al (2012) The Shigella flexneri effector OspI deamidates UBC13 to dampen the inflammatory response. Nature 483: 623–626. 22407319 10.1038/nature10894

[18] Rohde JR, Breitkreutz A, Chenal A, Sansonetti PJ, Parsot C (2007) Type III secretion effectors of the IpaH family are E3 ubiquitin ligases. Cell Host Microbe 1: 77–83. 18005683 10.1016/j.chom.2007.02.002

[19] Singer AU, Rohde JR, Lam R, Skarina T, Kagan O, et al (2008) Structure of the Shigella T3SS effector IpaH defines a new class of E3 ubiquitin ligases. Nat Struct Mol Biol 15: 1293–1301. 18997778 10.1038/nsmb.1511 PMC2764551

[20] Zhu Y, Li H, Hu L, Wang J, Zhou Y, et al (2008) Structure of a Shigella effector reveals a new class of ubiquitin ligases. Nat Struct Mol Biol 15: 1302–1308. 18997779 10.1038/nsmb.1517

[21] Ashida H, Kim M, Schmidt-Supprian M, Ma A, Ogawa M, et al.. (2010) A bacterial E3 ubiquitin ligase IpaH9.8 targets NEMO/IKKgamma to dampen the host NF-kappaB-mediated inflammatory response. Nat Cell Biol 12: 66–73; sup 61–69. 10.1038/ncb2006 PMC3107189 20010814

[22] Buchrieser C, Glaser P, Rusniok C, Nedjari H, D’Hauteville H, et al (2000) The virulence plasmid pWR100 and the repertoire of proteins secreted by the type III secretion apparatus of Shigella flexneri. Mol Microbiol 38: 760–771. 11115111 10.1046/j.1365-2958.2000.02179.x

[23] Le Gall T, Mavris M, Martino MC, Bernardini ML, Denamur E, et al (2005) Analysis of virulence plasmid gene expression defines three classes of effectors in the type III secretion system of Shigella flexneri. Microbiology 151: 951–962. 15758240 10.1099/mic.0.27639-0

[24] Kim DW (2005) The Shigella flexneri effector OspG interferes with innate immune responses by targeting ubiquitin-conjugating enzymes. Proceedings of the National Academy of Sciences 102: 14046–14051. 10.1073/pnas.0504466102 PMC1236552 16162672

[25] Hurley JH, Lee S, Prag G (2006) Ubiquitin-binding domains. Biochem J 399: 361–372. 17034365 10.1042/BJ20061138 PMC1615911

[26] Husnjak K, Dikic I (2012) Ubiquitin-binding proteins: decoders of ubiquitin-mediated cellular functions. Annu Rev Biochem 81: 291–322. 22482907 10.1146/annurev-biochem-051810-094654

[27] Hochstrasser M (2009) Origin and function of ubiquitin-like proteins. Nature 458: 422–429. 19325621 10.1038/nature07958 PMC2819001

[28] van der Veen AG, Ploegh HL (2012) Ubiquitin-like proteins. Annu Rev Biochem 81: 323–357. 22404627 10.1146/annurev-biochem-093010-153308

[29] Cheek S, Zhang H, Grishin NV (2002) Sequence and structure classification of kinases. J Mol Biol 320: 855–881. 12095261 10.1016/s0022-2836(02)00538-7

[30] Taylor SS, Radzio-Andzelm E (1994) Three protein kinase structures define a common motif. Structure 2: 345–355. 8081750 10.1016/s0969-2126(00)00036-8

[31] Hemrajani C, Berger CN, Robinson KS, Marches O, Mousnier A, et al (2010) NleH effectors interact with Bax inhibitor-1 to block apoptosis during enteropathogenic Escherichia coli infection. Proc Natl Acad Sci U S A 107: 3129–3134. 20133763 10.1073/pnas.0911609106 PMC2840288

[32] Chen G, Porter MD, Bristol JR, Fitzgibbon MJ, Pazhanisamy S (2000) Kinetic mechanism of the p38-alpha MAP kinase: phosphoryl transfer to synthetic peptides. Biochemistry 39: 2079–2087. 10684658 10.1021/bi9919495

[33] Mendelow M, Prorok M, Salerno A, Lawrence DS (1993) ATPase-promoting dead end inhibitors of the cAMP-dependent protein kinase. J Biol Chem 268: 12289–12296. 8509366

[34] Young PR, McLaughlin MM, Kumar S, Kassis S, Doyle ML, et al (1997) Pyridinyl imidazole inhibitors of p38 mitogen-activated protein kinase bind in the ATP site. J Biol Chem 272: 12116–12121. 9115281 10.1074/jbc.272.18.12116

[35] Ward NE, O’Brian CA (1992) The intrinsic ATPase activity of protein kinase C is catalyzed at the active site of the enzyme. Biochemistry 31: 5905–5911. 1535219 10.1021/bi00140a029

[36] Juris SJ, Rudolph AE, Huddler D, Orth K, Dixon JE (2000) A distinctive role for the Yersinia protein kinase: actin binding, kinase activation, and cytoskeleton disruption. Proc Natl Acad Sci U S A 97: 9431–9436. 10920208 10.1073/pnas.170281997 PMC16881

[37] Coaker G, Falick A, Staskawicz B (2005) Activation of a phytopathogenic bacterial effector protein by a eukaryotic cyclophilin. Science 308: 548–550. 15746386 10.1126/science.1108633

[38] Mittal R, Peak-Chew SY, Sade RS, Vallis Y, McMahon HT (2010) The acetyltransferase activity of the bacterial toxin YopJ of Yersinia is activated by eukaryotic host cell inositol hexakisphosphate. J Biol Chem 285: 19927–19934. 20430892 10.1074/jbc.M110.126581 PMC2888404

