# Supplementary figures and images for: The Shigella Type Three Secretion System Effector OspG Directly and Specifically Binds to Host Ubiquitin for Activation
Source: PLoS One. 2013 Feb 28;8(2):e57558. doi: 10.1371/journal.pone.0057558 (PMC3585378; doi:10.1371/journal.pone.0057558)

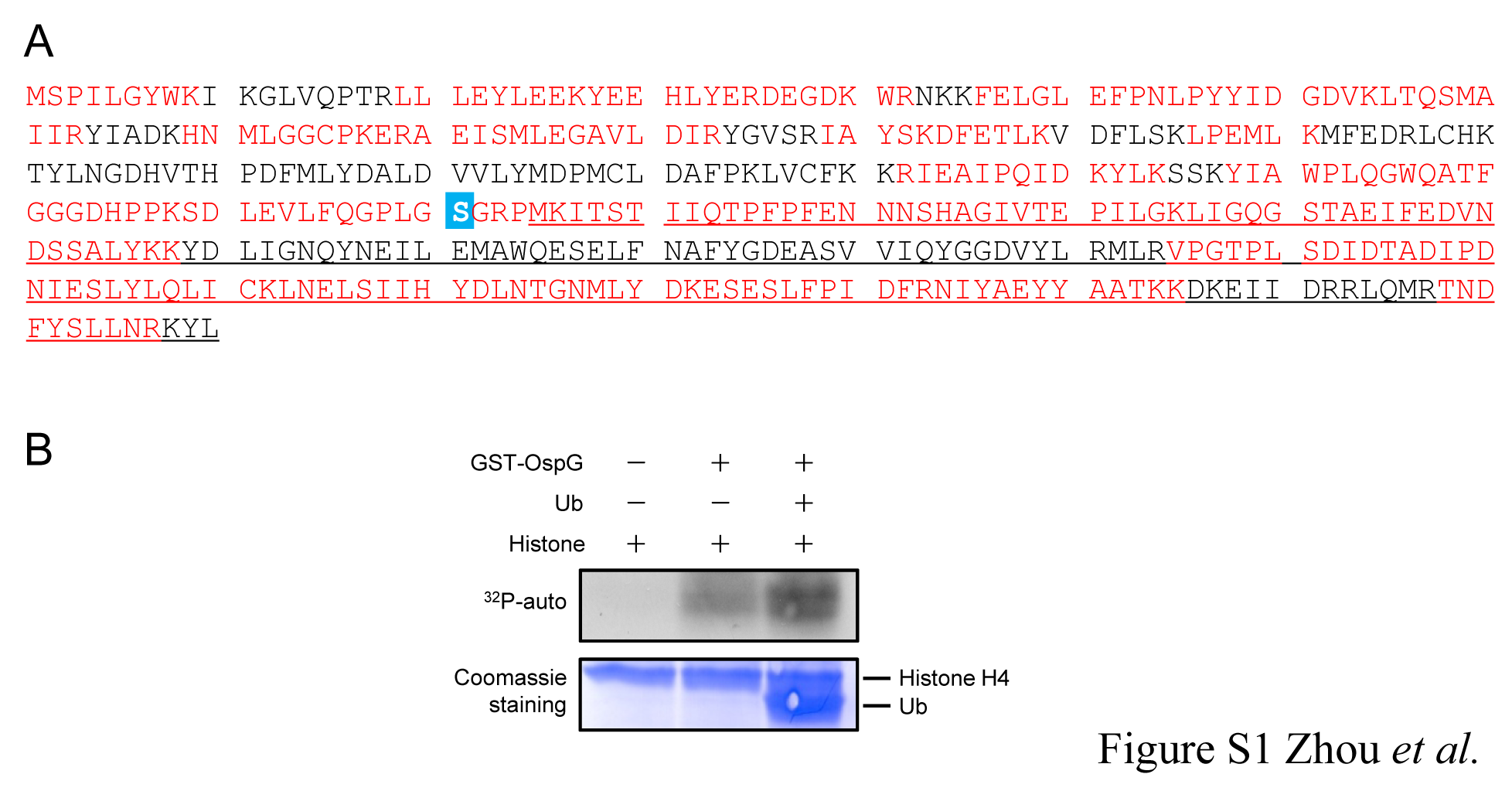

Supplement: Figure S1 — Autophosphorylation site in GST-OspG and kinase assay of OspG. (A) Shown is the amino acid sequence of recombinant GST-OspG protein used in the kinase assay and subjected to mass spectrometry analysis. Residues in red correspond to the tryptic peptide sequence identified by mass spectrometry analysis. The sequence of OspG is underlined. The serine residue identified as the phosphorylation site is shown on a blue background. (B) OspG kinase assay using commercial histones as the artificial substrates. The assay was performed in the absence or presence of ubiquitin. (TIF) [file pone.0057558.s001.tif]

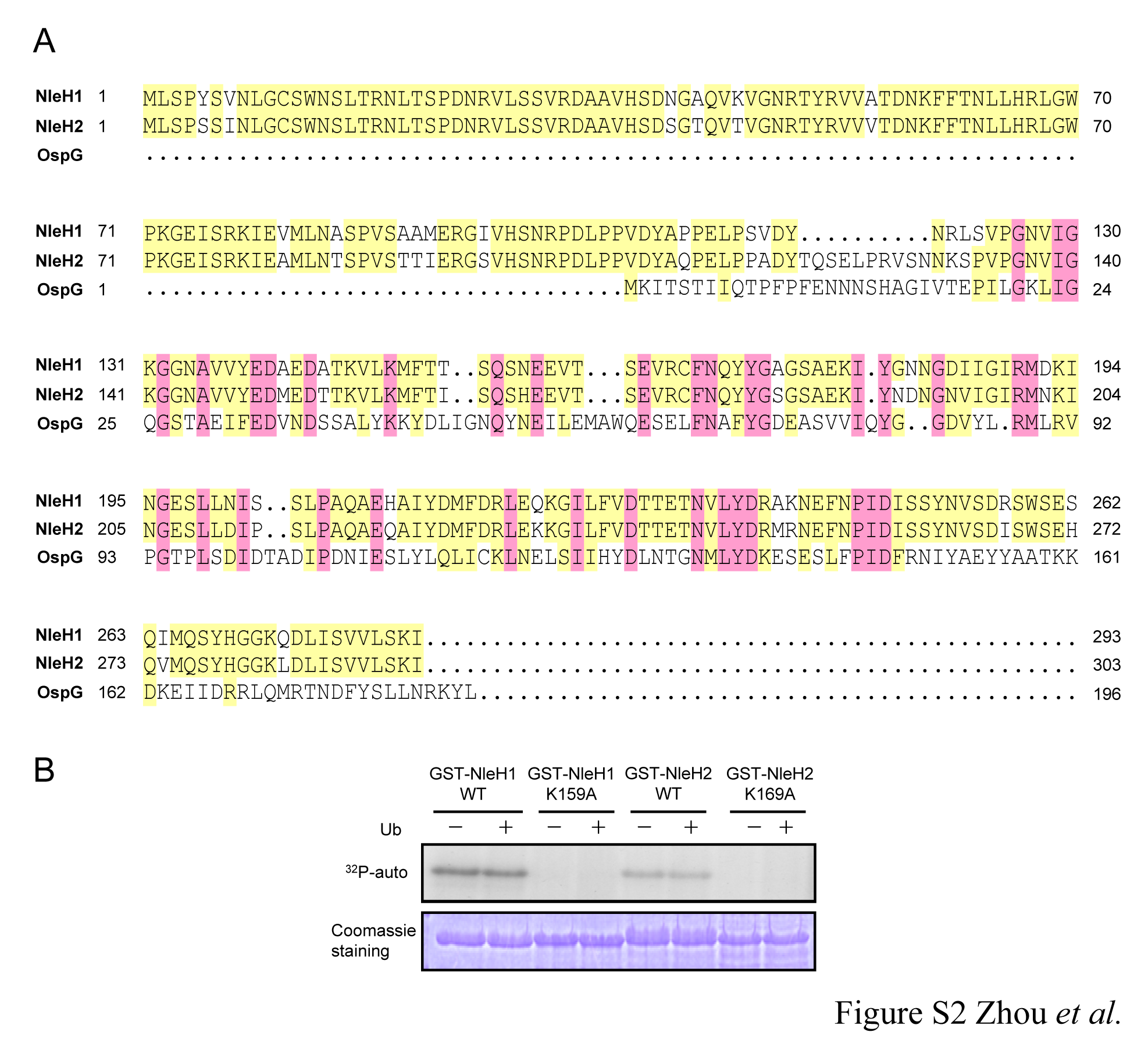

Supplement: Figure S2 — Ubiquitin does not stimulate the kinase activity of OspG-like effector NleH1/2. (A) Sequence alignment of Shigella flexneri OspG with NleH1/2 from enteropathogenic Escherichia coli O55:H7 (strain CB9615). The protein names are listed on the left of the alignment. Conserved residues are marked in pink, and similar residues are shown in green. (B) The kinase assay of GST-NleH1 (WT and K159A mutant) and GST-NleH2 (WT and K169A mutant) in the absence or presence of ubiquitin. (TIF) [file pone.0057558.s002.tif]
